# DHX9 Strengthens Atherosclerosis Progression By Promoting Inflammation in Macrophages

**DOI:** 10.1007/s10753-023-01836-z

**Published:** 2023-06-16

**Authors:** Ning Huangfu, Hongchuang Ma, Mengyun Tian, Jie Zhang, Yong Wang, Zhenwei Li, Xiaomin Chen, Hanbin Cui

**Affiliations:** 1 Department of Cardiology, Ningbo First Hospital, Ningbo, 315000 China; 2 Key Laboratory of Precision Medicine for Atherosclerotic Diseases of Zhejiang Province, Ningbo, 315000 China; 3 Clinical Medicine Research Centre for Cardiovascular Disease of Ningbo, Ningbo, 315000 China; 4 School of Medicine, Ningbo University, Ningbo, 315000 China

**Keywords:** atherosclerosis, DExH-box helicase 9, macrophage, inflammation, p65.

## Abstract

Atherosclerosis (AS) is the main cause of cerebrovascular diseases, and macrophages play important roles in atherosclerosis. DExH-Box helicase 9 (DHX9), as a member of DExD/H-box RNA helicase superfamily II, is identified as an autoantigen in the sera of systemic lupus erythematosus patients to trigger inflammation. The aim of this study was to investigate whether DHX9 is involved in AS development, especially in macrophages-mediated-inflammatory responses. We find that DHX9 expression is significantly increased in oxLDL or interferon-γ-treated macrophages and peripheral blood mononuclear cells (PBMCs) from patients with coronary artery disease (CAD). Knockdown of DHX9 inhibits lipid uptake and pro-inflammatory factors expression in macrophages, and ameliorates TNF-α-mediated monocyte adhesion capacity. Furthermore, we find that oxLDL stimulation promotes DHX9 interaction with p65 in macrophages, and further enhances the transcriptional activity of DHX9-p65-RNA Polymerase II complex to produce inflammatory factors. Moreover, using ApoE -/- mice fed with western diet to establish AS model, we find that knockdown of DHX9 mediated by adeno-associated virus-Sh-DHX9 through tail vein injection evidently alleviates AS progression *in vivo*. Finally, we also find that knockdown of DHX9 inhibits p65 activation, inflammatory factors expression, and the transcriptional activity of p65-RNA Polymerase II complex in PBMCs from patients with CAD. Overall, these results indicate that DHX9 promotes AS progression by enhancing inflammation in macrophages, and suggest DHX9 as a potential target for developing therapeutic drug.

## INTRODUCTION

Atherosclerosis (AS), as the major underlying pathology of cardiovascular diseases, is a chronic immune-inflammatory disease characterized by accumulation of plaque in blood vessels [1, 2]. In the early stage of atherogenesis, monocytes in the blood are recruited by Low-density lipoprotein (LDL) and infiltrated into the intima [3, 4]. Then, monocytes are differentiated into macrophages, which uptake excessive oxidized LDL (ox-LDL) to form foam cells and produce pro-inflammatory cytokines, eventually leading the formation of plaque in arteries [4–6]. Besides LDL, other factors, such as proatherogenic sialidases, desialylated LDL, mutations of mitochondrial DNA, mitochondrial dysfunction, and genders, are also involved in AS development and affect clinical outcomes [7–9]. Up to now, there is still short of effective therapeutic drugs for AS. Thus, exploring the pathological mechanism of macrophage-mediated inflammatory responses in AS is essential for developing drugs for AS therapy.

DExH-Box helicase 9 (DHX9), as a member of DExD/H-box RNA helicase superfamily II, plays important roles in regulating RNA processing and transport, microRNA processing, DNA replication and transcription [10]. Nowadays, DHX9 is found to be involved in the development of several cancers and in autoimmune diseases [10]. For example, DHX9 promotes glioblastomas development by interacting with epidermal growth factor receptor (EGFR) to activate transcription of EGFR-responsive genes [11]. Membrane-associated ring-CH-type finger (MARCH) protein promotes papillary thyroid cancer development by ubiquitinating DHX9 [12]. DHX9 is identified as an autoantigen in the sera of systemic lupus erythematosus patients to trigger inflammation and complications such as skin rashes, and atherosclerosis [13, 14]. In addition, DHX9 has been reported to induce expressions of pro-inflammatory cytokines and interferons in myeloid dendritic cells by interacting with mitochondrial antiviral signaling protein (MAVS) to sense double-stranded RNA [15, 16]. However, whether DHX9 is involved in AS development, especially in macrophages-mediated inflammatory responses remains unknown.

In this study, we investigated the roles of DHX9 on macrophage-mediated inflammatory responses in AS *in vitro* and *in vivo*. Our study firstly finds that DHX9 expression is significantly increased in oxLDL or interferon-γ-treated macrophages and peripheral blood mononuclear cells (PBMCs) from patients with coronary artery disease (CAD). Besides, we find that DHX9 enhances the transcriptional activity of p65-RNA Polymerase II complex to produce inflammatory factors by interacting with p65. Moreover, knockdown of DHX9 evidently alleviates AS progression *in vivo* using AS mice model, and significantly decreases the inflammatory factor expressions in PBMCs from CAD patients.

## MATERIALS AND METHODS

### Cell Culture

Human umbilical vein endothelial cells (HUVECs), and human monocyte-like cell line (THP-1) were cultured in HUVEC specific medium (PROCELL, CHINA, Ham's F-12 K, 0.1 mg/mL Heparin, 0.03–0.05 mg/mL ECGs, 1% P/S) or RPMI-1640 (Gibco, USA) medium supplied with 10% fetal bovine serum (Gibco, USA). The THP-1 cells were stimulated with phorbol ester (PMA) (100 nM, sigma, USA) to differentiate macrophage-like sticky cells for later experiments. Peritoneal macrophages were isolated as previously described [14]. Human peripheral blood sample from healthy volunteers (HV) or patients with coronary artery disease (CAD) were obtained from the Ningbo First Hospital, which was allowed by the ethics committee of Ningbo First Hospital. Human peripheral blood-derived mononuclear cells, separated from human peripheral blood with human lymphocyte separation medium (Sigma), were cultured in RPMI-1640 medium containing 10% fetal bovine serum, and then were differentiated into macrophages in the presence of 50 ng/ml of recombinant human GM-CSF (Peprotech) for 4 days, as previously described [17–19]. IFN-γ was purchased from BioLegend. ox-LDL and recombinant human TNF-α protein were purchased from Beyotime.

### Western Blotting

Western blotting was performed as previously described [17, 20]. Briefly, cells were collected, lysed, and then quantified with a BCA kit (Beyotime, China). 20 μg of protein was loaded into a SDS gel, followed by electronic transfer onto PVDF membranes (Millipore, USA), and then incubated with primary antibodies against DHX9 (No.17721–1-AP, Proteintech), STAT1 (No.10144–2-AP, Proteintech), STAT1 phospho Ser727 (p-STAT1, No.39634, Active motif), p38 (No.14064–1-AP, Proteintech), Phospho-P38 MAPK (Thr180/Tyr182) (p-p38, No.28796–1-AP, Proteintech), JNK (No.66210–1-Ig, Proteintech), Phospho-JNK (Tyr185) (p-JNK, No.80024–1-RR, Proteintech), ERK1/2 (No.11257–1-AP, Proteintech), Phospho-ERK1/2 (Thr202/Tyr204) (p-ERK1/2, No.80031–1-RR, Proteintech), p65 (No.66535–1-Ig, Proteintech), p65 (phospho S536) (p-p65, sc-136548, Santa Cruz), GAPDH (ab8245, Abcam), flag (ab205606, Proteintech), or Lamin B1 (No.66095–1-Ig, Proteintech). After incubating with the secondary antibodies, the protein bands were detected using an ECL kit (Pierce, USA). The experiment was repeated three times independently.

### Immunofluorescence

Immunofluorescence was performed as previously described [17, 20]. Briefly, after treatments, cells were fixed, permeabilized, and stained with primary antibodies against DHX9 (No.17721–1-AP, Proteintech), p65 (No.66535–1-Ig, Proteintech), p65 (phospho S536) (p-p65, sc-136548, Santa Cruz), F4/80 (No.28463–1-AP, Proteintech) at 4 °C overnight. After incubating with the second antibodies, cellular nuclei were stained with DAPI (0.5 μg/ml, Cell signaling) and then imaged by a Carl Zeiss LSM 800 confocal.

### Plasmids, Small Interfering RNAs (siRNAs) and Adeno-associated Virus

siRNA of DHX9 was synthetized and purchased from Shanghai GenePharma Co., Ltd (China). pCMV3-N-FLAG-DHX9 plasmid was obtained from Sino Biological Co., Ltd (China). siRNAs and plasmid were transfected with jetPRIME transfection reagent (polyplus, FRANCE), as previously described [17]. Adeno-associated virus serotype 9 (AAV)-GP-3-sh-DHX9 (AAV-sh-DHX9) and control AAV (AAV-sh-NC) were synthetized and purchased from GenePharma.

### Real Time-PCR (RT-PCR)

RT-PCR was performed as previously described [17]. Primers used for RT-PCR were as follows: human DHX9, F: CGAACCATCTCAGCGACAAAA, R: TGAGGTCCATGCTTATTTGCTC; human TNFα, F: CCTCTCTCTAATCAGCCCTCTG, R: GAGGACCTGGGAGTAGATGAG; human IL1β, F: ATGATGGCTTATTACAGTGGCAA, R: GTCGGAGATTCGTAGCTGGA; human IL6, F: ACTCACCTCTTCAGAACGAATTG, R: CCATCTTTGGAAGGTTCAGGTTG; mouse IL6, F: TAGTCCTTCCTACCCCAATTTCC, R: TTGGTCCTTAGCCACTCCTTC; mouse TNFα, F: CCCTCACACTCAGATCATCTTCT, R: GCTACGACGTGGGCTACAG; human GAPDH, F: GGAGCGAGATCCCTCCAAAAT, R: GGCTGTTGTCATACTTCTCATGG; Mouse GAPDH, F: AGGTCGGTGTGAACGGATTTG, R: TGTAGACCATGTAGTTGAGGTCA.

### Enzyme-linked Immunosorbent Assay (ELISA)

The TNFα or IL6 protein expression in the Serum of mice was measured using the mouse TNF alpha ELISA Kit (ab208348, Abcam) or the mouse IL-6 ELISA Kit (ab222503, Abcam), according to the manufacturer’s instructions.

### DiI-ox-LDL Uptake Analysis

THP-1-derived macrophages were transfected with control siRNA (si-NC) or si-DHX9 for 48 h before exposure to 50 μg/mL DiI-ox-LDL (20609ES76, YEASEN) for 30 min at 37 °C. Then, the cells were fixed, permeabilized, and stained with primary antibodies against DHX9 (No.17721–1-AP, Proteintech), followed by staining with the second antibodies. Cellular nuclei were stained with DAPI (0.5 μg/ml, Cell signaling) and then imaged by a Carl Zeiss LSM 800 confocal.

### Monocyte-Endothelial Cell Adhesion Assay

The monocyte-endothelial cell adhesion assay was performed following previously described [21, 22]. Briefly, HUVECs (1.5 × 10^5^ cells/mL) in the 96-well plates were treated with 10 ng/mL TNF-α (P5318, Beyotime) for 5 h. After transfection with si-NC or si-DXH9 for 48 h, THP-1 cells were fluorescently labeled with 2 μM calcein AM (Sigma-Aldrich) for 15 min at 37 °C. Then, the HUVECs and THP-1 cells were co-incubated for 1 h. After washing three times with PBS, the cells were imaged using a Carl Zeiss LSM 800 confocal.

### Flow Cytometry Assay

After transfection with si-NC or si-DXH9 for 48 h, the apoptotic rates or the cell cycle of THP-1 cells were analyzed with the Annexin V-PE Apoptosis Detection Kit (C1065M, Beyotime), or the Cell Cycle Analysis Kit (C1052, Beyotime) by a flow cytometer (Beckman, USA).

### Bioinformatic Analysis

DHX9 mRNA expressions in the mononuclear phagocytes were analyzed using online analysis website (https://gustaveroussy.github.io/FG-Lab/). The website is a platform to provide access to databases used in our integrative studies. This tool in the website allows exploration of metadata and gene expression profiles across tissues, conditions and clusters, and the data is publicly available through an online interactive tool via the CellXGene.

### Separation of Cytoplasmic and Nuclear Fractions

After treatment, the cytoplasmic and nuclear fractions of THP-1 cells were separated by the Nuclear and Cytoplasmic Protein Extraction Kit (P0027, Beyotime), according to the manufacturer’s manual.

### Protein Immunoprecipitation (Co-IP)

Co-IP was performed as previously described [17, 23]. Protein samples were immunoprecipitated with antibodies against DHX9 (No.17721–1-AP, Proteintech). Then, IP production was performed with Western blotting.

### Chromatin Immunoprecipitation (ChIP) Assays

ChIP assays were performed with the SimpleChIP^®^ Enzymatic Chromatin IP Kit (Cell Signaling Technology, USA) as previously described [17, 23]. Chromatin samples were immunoprecipitated with antibodies against a negative control normal IgG, p65 (ab32536, Abcam), anti-RNA polymerase II (ab264350, Abcam) or DHX9 (No.17721–1-AP, Proteintech), respectively. Then, IP production was performed with RT-qPCR. For the ChIP-re-ChIP experiments, the supernatant containing anti-DHX9, p65, or RNA polymerase II antibody-immunoprecipitated cross-linked protein-DNA complexes was further immunoprecipitated with magnetic beads coated anti-p65, DHX9, or RNA polymerase II antibody. Then, the immunoprecipitated DNA was purified for quantitative PCR analyses. The primers of IL-6 promoter were as following F: AGACCAGTGATTTTCACCAGG, R: TGGCATGAGCTGAGGGTTATTGC.

### Animal Experiments

ApoE (−/−) mice (male, 7 week-old, n = 12) were provided and housed in the Model Animal Research Center of ServiceBio (Wuhan, China) at an ambient temperature of 20–24 °C with 40–70% relative humidity under a 12:12 h light–dark cycle. All mouse experiments were conducted after approval by the ethics committee of Ningbo First Hospital. The mice were randomly divided into the control group (n = 6), and DHX9-downregulated group (n = 6), which were injected with 10^11^ VG AAV-sh-NC, or AAV-Sh-DHX9 through tail vein, respectively. One week later, mice were fed a high fat diet (16.9% fat, 1.3% cholesterol, 21.1% crude protein, and 46.5% carbohydrates) for 12 weeks to construct a mouse model of atherosclerosis. Once the model of atherosclerosis was established (20 weeks old), mice were sacrificed by CO_2_ asphyxiation. Aorta from the ascending aorta to the arteria iliaca communis was isolated and stained using oil red (Beyotime, China). Atherosclerotic lesion sizes were assessed using Image Pro Plus 6.0 and expressed as the percentage of plaque area relative to the total intimal area.

### Human Peripheral Blood Samples

A total of 45 volunteers were recruited for this study, of which 30 were patients with coronary heart disease (15 males and 15 females) and 15 were healthy people (7 males and 8 females) matching the age and sex of the coronary heart disease group. All the patients were diagnosed with coronary heart disease by coronary angiography (at least 1 vessel ≥ 80% stenosis) and healthy people (0% stenosis) with age range of age 59.6 ± 8.8 and 58.0 ± 8.3 years, respectively. 19 patients were diagnosed with 1 vessel stenosis, 5 with 2 vessels stenosis and 6 with 3 vessels stenosis. Human sample collection and application were supervised by of the ethics committee of The First Affiliated Hospital of NINGBO university, and adhered to the principles of the Declaration of Helsinki and Istanbul. All individuals signed informed consent for the use of clinical specimens in the present study. 

### Statistics Analysis

Data throughout the paper are expressed as mean ± SD. Statistical differences were calculated using unpaired two-tailed Student’s t test or one-way ANOVA with Bonferroni correction for multiple comparisons. A probability of p < 0.05 was considered statistically significant. Ns not significant.

## RESULTS

### DHX9 Expression is Significantly Increased in OxLDL or Interferon-γ-treated Macrophages and Peripheral Blood Mononuclear Cells From Patients With Coronary Artery Disease

**Fig. 1:**
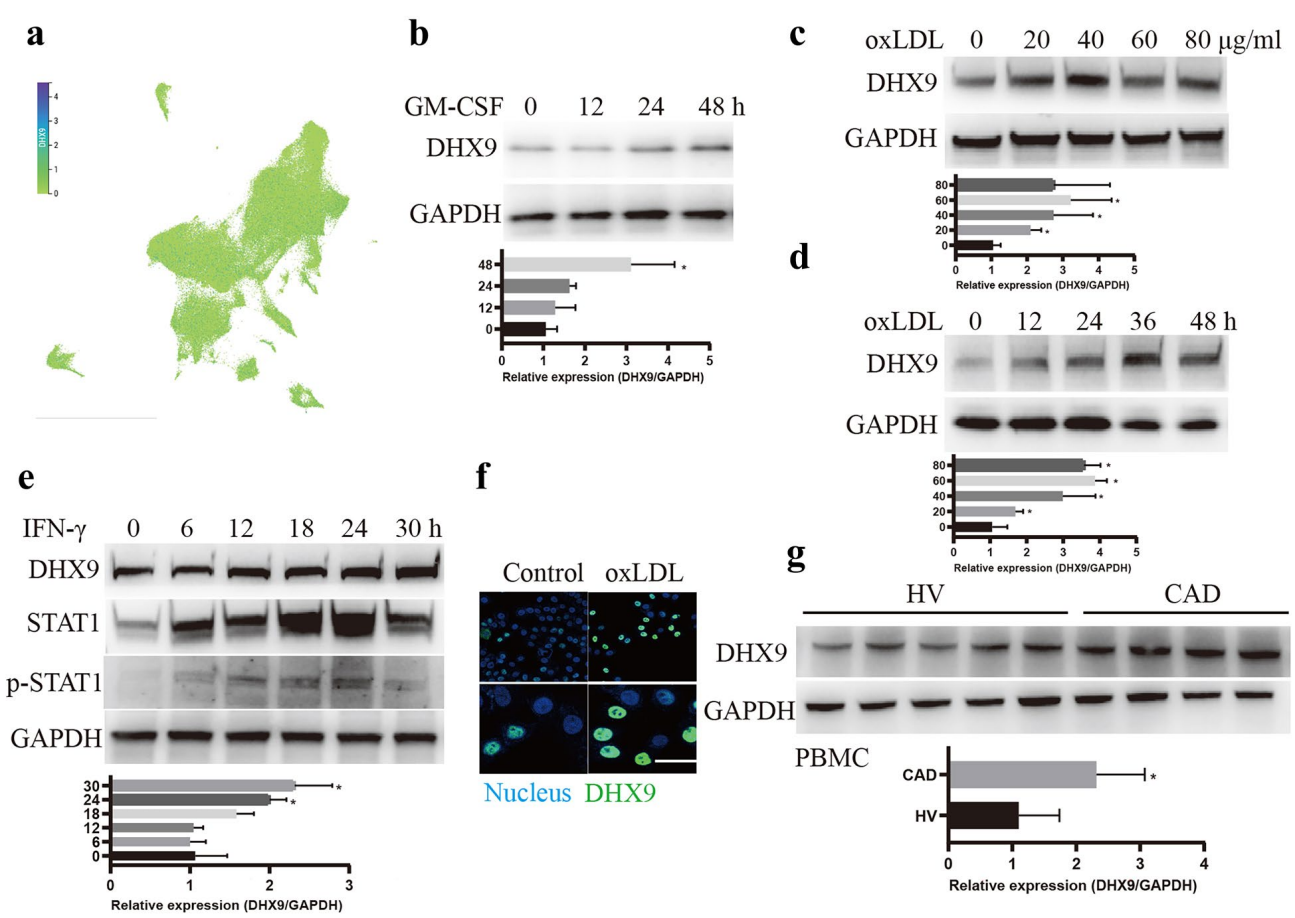
DHX9 expression significantly increased in oxLDL or interferon-γ-treated macrophages and peripheral blood mononuclear cells from patients with coronary artery disease. **a** Single-cell RNA analysis of DHX9 mRNA expressions in mononuclear phagocytes (MNPs) a public MNP single-cell RNA compendium. **b** Western blot analysis of DHX9 expressions PBMCs from health volunteers stimulated with 50 ng/ml of recombinant human GM-CSF for different hours. **c** Western blot analysis of DHX9 expressions in the THP-1-derived macrophages stimulated with oxLDL for 24 h. **d** Western blot analysis of DHX9 expressions in the THP-1-derived macrophages stimulated with 40 μg/ml oxLDL for different hours. **e** Western blot analysis of DHX9, STAT1, p-STAT1 expressions in the THP-1-derived macrophages stimulated with 20 ng/mL IFN-γ for different hours. GAPDH was used as a loading control. **f** Immunofluorescence analysis of DHX9 cellular distribution in macrophages treated with or without 40 μg/mL oxLDL for 24 h. Scale bars, 10 μm. **g** Western blot analysis of DHX9 expressions in PBMCs isolated from health volunteers (HV) or patients with (CAD). GAPDH was used as a loading control.

To explore whether DHX9 is involved in AS development, especially in macrophages-mediated inflammatory responses, we firstly analyzed the expression of DHX9 mRNA in mononuclear phagocytes (MNPs), including dendritic cells, monocytes, and macrophages in blood using a public MNP single-cell RNA compendium, and found that DHX9 is expressed in all the cellular subsets of MNPs (Fig. 1a). Then, we examined the DHX9 expressions in PBMCs from healthy volunteers (HV) stimulated with granulocyte–macrophage colony-stimulating factor (GM-CSF) to induce monocytes to differentiate into macrophages [24], and found that DHX9 expression is gradually increased in a time-dependent manner in monocytes under GM-CSF stimulation (Fig. 1b). Subsequently, we examined the DHX9 expressions in THP-1-derived macrophages treated with oxLDL, and found that oxLDL stimulation evidently promoted DHX9 expressions in a dose/time dependent manner (Fig. 1c, d). Furthermore, we further examined the DHX9 expressions in M1 macrophages derived from THP-1 cells stimulated with interferon-γ (IFN-γ), which express pro-inflammatory factors [25], and found that DHX9 expression is gradually increased in a time-dependent manner in macrophages under IFN-γ stimulation, combined with the gradually increased expressions of M1 macrophage makers, signal transducer and activator of transcription 1 (STAT1) and phosphorylated STAT1 (p-STAT1) (Fig. 1e). Moreover, immunofluorescent staining also confirmed that oxLDL stimulation evidently increased DHX9 expressions (Fig. 1f). Finally, we examined the DHX9 expression level in PBMCs from HV or patients with CAD. As expected, DHX9 protein expression levels are indeed higher in PBMCs from patients with CAD, compared to those in HV (Fig. 1g). Overall, these results implicate that DHX9 expression is significantly increased in oxLDL or IFN-γ-treated macrophages and PBMCs from patients with CAD.

### Knockdown of DHX9 Inhibits Lipid Uptake and Pro-inflammatory Factor Expressions of Macrophages, and Ameliorates TNF-α-mediated Monocyte Adhesion Capacity

**Fig. 2:**
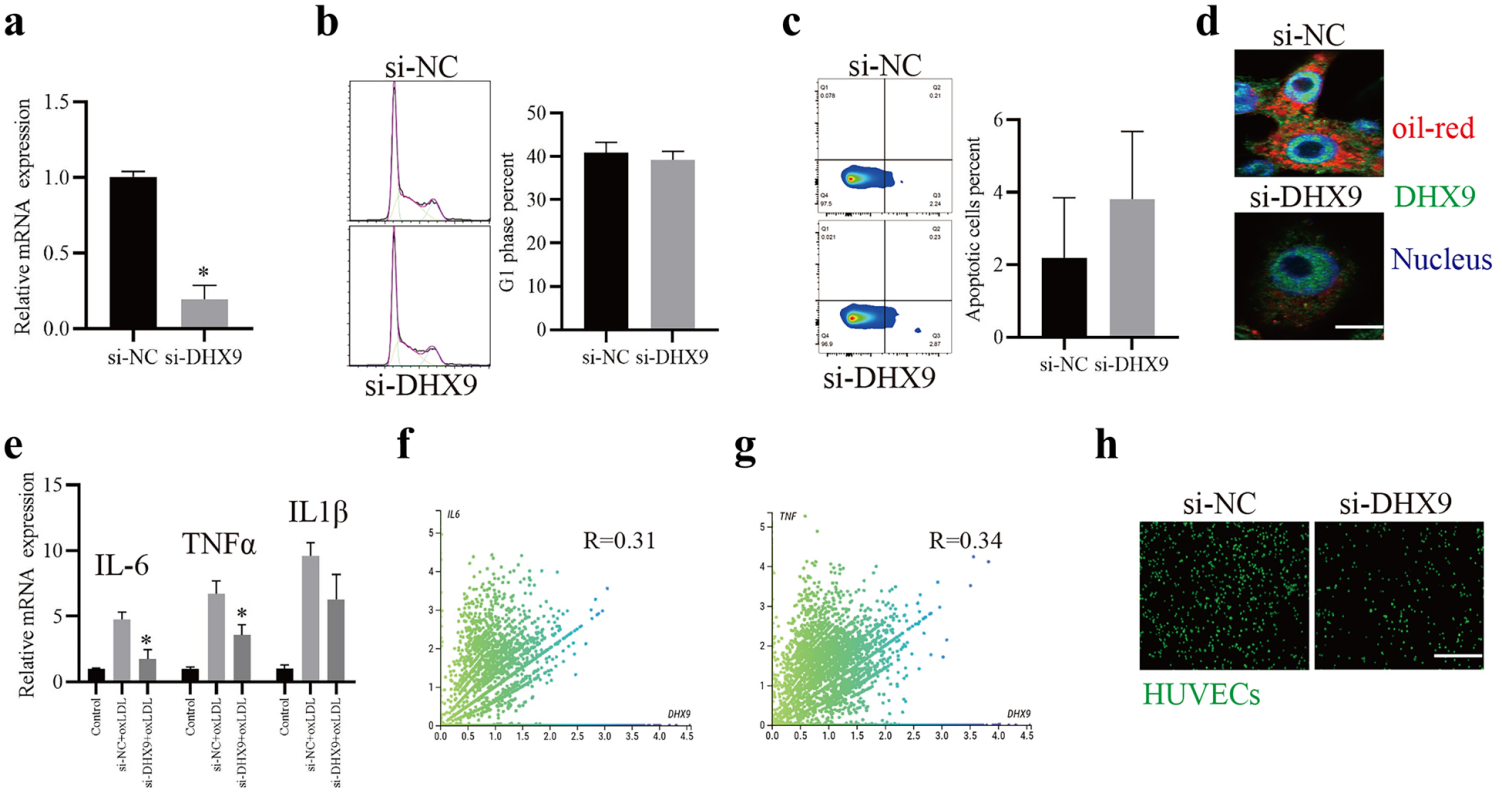
Knockdown of DHX9 inhibits lipid uptake and pro-inflammatory factor expressions of macrophages, and ameliorates TNF-α-mediated monocyte adhesion capacity. **a** qPCR detection of IL-6 and TNF mRNAs in THP-1-derived macrophages transfected with control siRNA (si-NC) or siRNA against DHX9 (si-DHX9) for 48 h. **b** Flow cytometry analysis of cell cycle of THP-1-derived macrophages transfected with control siRNA (si-NC) or siRNA against DHX9 (si-DHX9) for 48 h. **c** Flow cytometry analysis of cell apoptosis of THP-1-derived macrophages transfected with control siRNA (si-NC) or siRNA against DHX9 (si-DHX9) for 48 h. **d** Immunofluorescence analysis of Dil-oxLDL (red) uptake of THP-1-derived macrophages transfected si-NC or si-DHX9 for 48 h. Scale bars, 5 μm. **e** qPCR detection of IL-1β, IL-6 and TNF-α mRNAs in THP-1-derived macrophages transfected with si-NC or si-DHX9 for 48 h and later incubated with 40 μg/mL oxLDL for 24 h. Data are represented as means ± SD (n = 3; * P < 0.05 vs. si-nc + oxLDL group). **f** and **g** Correlation analysis of DXH9 and IL-6 or TNFα gene expressions in monocytes. **h** Representative images of the attachment of THP-1 cells transfected si-NC or si-DHX9 to HUVECs. Scale bars, 160 μm. Data are represented as means ± SD. n = 3; Statistical differences were calculated using unpaired two-tailed Student’s t test. *P < 0.05.

Considering that macrophages-mediated lipid uptake and pro-inflammatory factor expressions functions essential roles in AS [26, 27], we further investigated whether DHX9 is related to these biological processes. Firstly, we found that knockdown of DHX9 did not significantly affect the cell viability, cell cycle or cell apoptosis of THP-1-derived macrophages (Fig. 2a to c). Furthermore, we found that knockdown of DHX9 notably suppressed THP-1-derived macrophages-mediated lipid uptake (Fig. 2d) and the expressions of pro-inflammatory factors, IL-6 and TNF-α, but not IL-1β (Fig. 2e). Moreover, we found that the expressions of DHX9 transcripts in monocytes are positively correlated to those of IL-6 (Fig. 2f) and TNF (Fig. 2g). Finally, considering that productions of pro-inflammatory factors is closely associated with recruiting monocytes to the endothelial cells in AS [22], we further examined that whether DHX9 affects TNF-α-induced monocyte THP-1 adhesion capacity on the HUVECs. As shown in Fig. 2h, knockdown of DHX9 notably ameliorates TNF-α-mediated monocyte adhesion capacity. Overall, these results implicate that knockdown of DHX9 inhibits lipid uptake and pro-inflammatory factor expressions of macrophages, and ameliorates TNF-α-mediated monocyte adhesion capacity.

### oxLDL Stimulation Promotes DHX9 Interaction With p65 in Macrophages

**Fig. 3:**
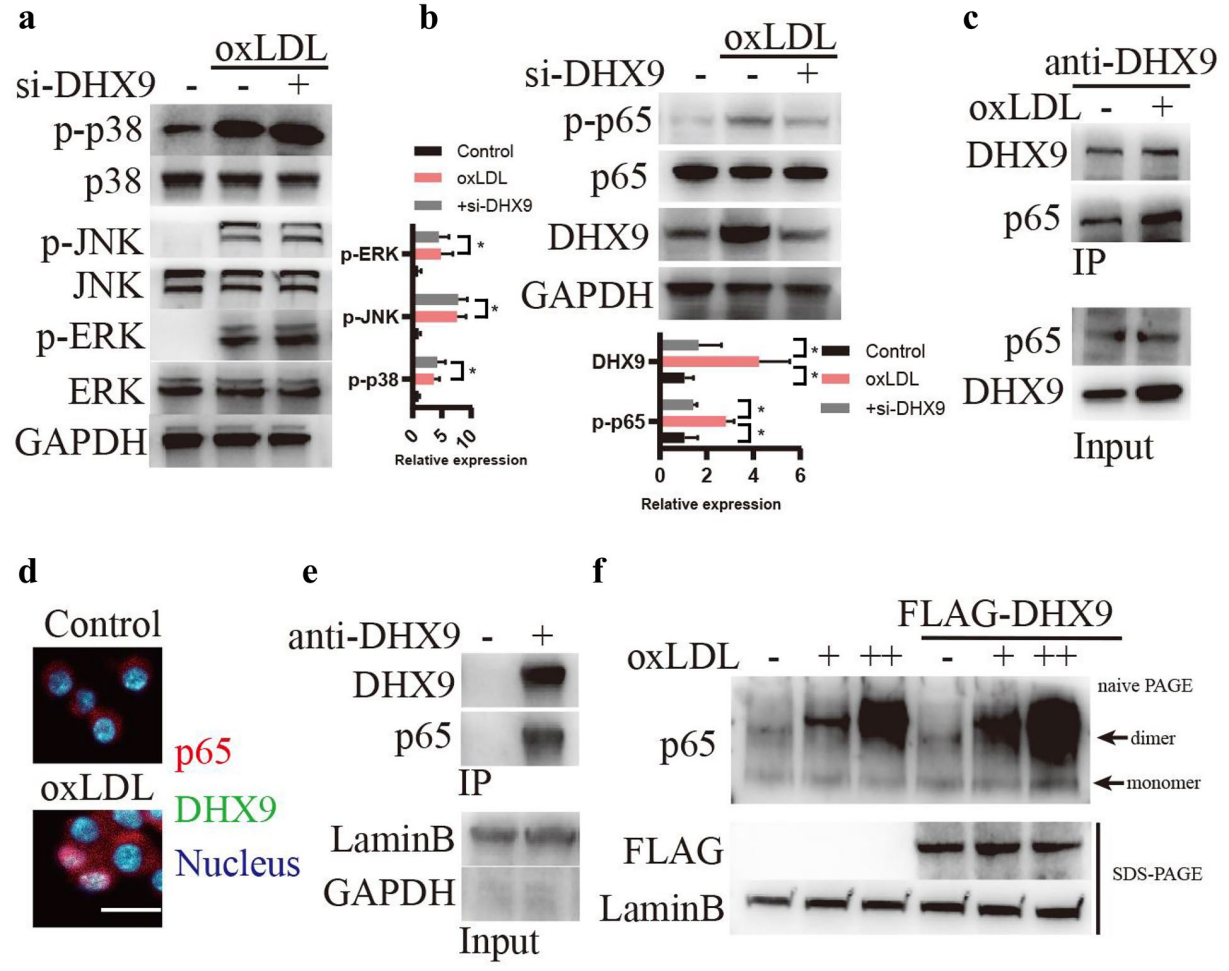
oxLDL stimulation promotes DHX9 interaction with p65 in macrophages. **a** Western blotting analysis of p38, JNK, ERK signaling in macrophages transfected with si-DHX9 or si-nc for 48 h and later incubated with 40 μg/mL oxLDL for 24 h. **b** Western blotting analysis of NF-κB signaling in macrophages transfected with si-DHX9 or si-nc for 48 h and later incubated with 40 μg/mL oxLDL for 24 h. **c** Co-IP detection of the interaction between DHX9 and p65 in macrophages treated with or without 40 μg/mL oxLDL for 24 h by using DHX9 antibody. **d** Immunofluorescence analysis of DHX9 and P65 expression in macrophages treated with or without 40 μg/mL oxLDL. Scale bars, 10 μm. **e** Co-IP detection of the interaction between DHX9 and p65 in the nuclear fractions of macrophages by using DHX9 antibody. **f** P65 dimer formation was detected using naïve PAGE when macrophages were transfected with or without FLAG-DHX9 and treated with or without oxLDL (-, 0 μg/mL oxLDL; +, 40 μg/mL oxLDL; ++, 80 μg/mL oxLDL).

Given that inflammatory response is generally triggered by activation of mitogen-activated protein kinase (MAPK) signaling, such as p38, Jun-NH(2)-terminal kinase (JNK), and extracellular signal-regulated kinase (ERK), and p65 nuclear factor kappa-light-chain-enhancer of activated B cells (NF-κB) signaling [28, 29], we then examined whether DHX9 promoting oxLDL-induced inflammatory response in macrophages is related to these signaling. As shown in Fig. 3a and b, oxLDL stimulation significantly increased the protein expressions of phosphorylated p38 (p-p38), p-JNK1/2, p-ERK and p-p65, whereas knockdown of DHX9 evidently inhibited the activation of p65, but not p38, JNK1/2, or ERK. We further examined whether DHX9 interacting with p65 in macrophages. As shown in Fig. 3c, oxLDL stimulation significantly increased DHX9 interaction with p65 by Co-IP. Furthermore, immunofluorescent staining showed that DHX9 and p65 were exclusively co-localized in the cellular nucleus (Fig. 3d), which was further confirmed by CO-IP assay to detect the interaction of DHX9 and p65 in the nuclear fractions (Fig. 3e). In addition, we also found that DHX9 promotes p65 dimer formation induced by oxLDL (Fig. 3f). Overall, these results implicate that oxLDL stimulation promotes DHX9 interaction with p65 in macrophages.

### oxLDL Stimulation Promotes the Transcriptional Activity of DHX9-p65-RNA Polymerase II Complex

**Fig. 4:**
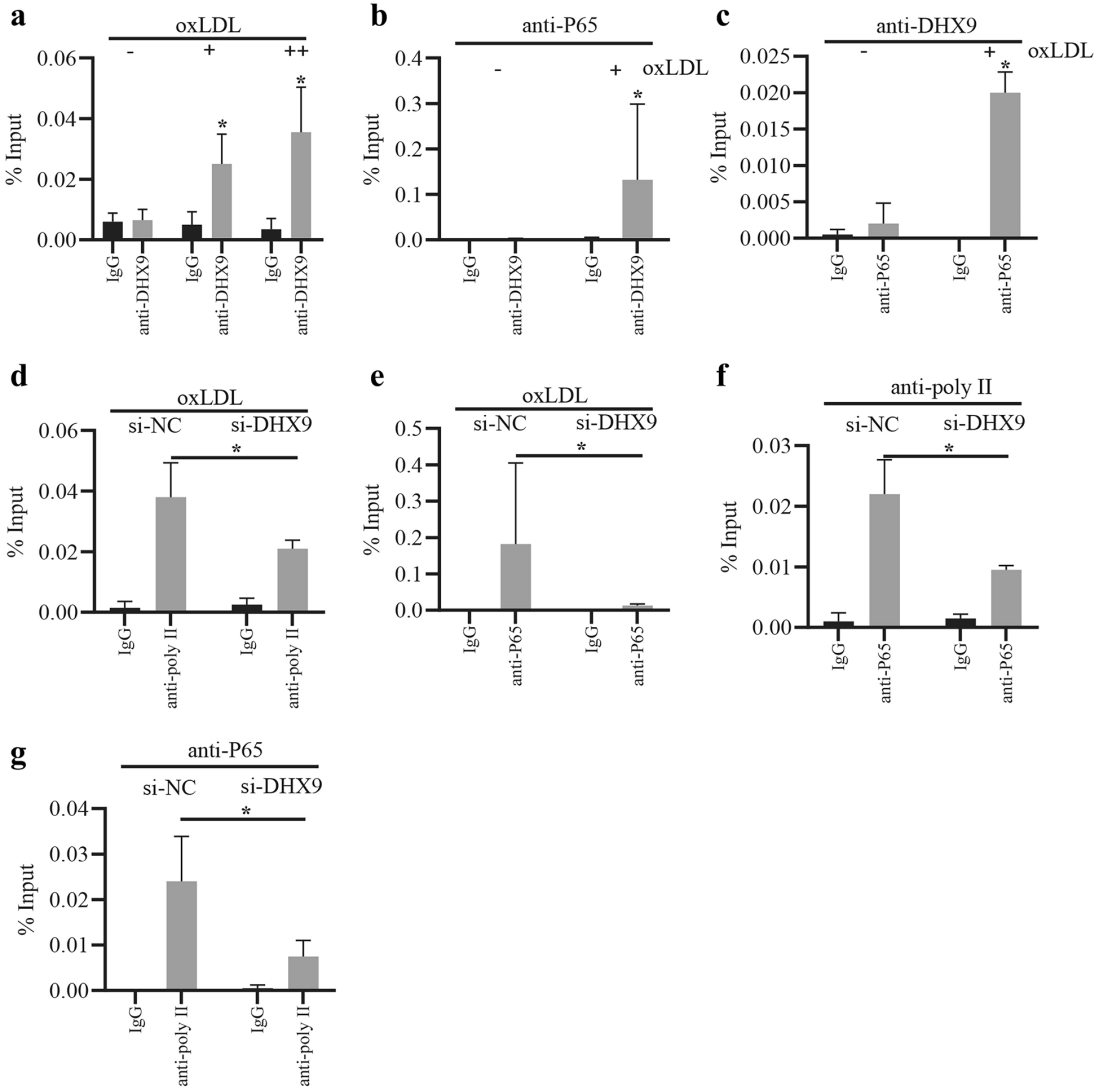
oxLDL stimulation promotes the transcriptional activity of DHX9-p65-RNA Polymerase II complex. **a** ChIP analysis of the binding of DHX9 to IL-6 promoter in macrophages treated with oxLDL (-, 0 μg/mL oxLDL; +, 40 μg/mL oxLDL; ++, 80 μg/mL oxLDL). IgG was used as the control of anti-DHX9. Data are represented as means ± SD (n = 3; *P < 0.05). **b** and **c** ChIP-re-ChIP analysis of the binding of DHX9-p65 complex to IL-6 promoter in macrophages treated with oxLDL. **d** ChIP analysis of the binding of RNA Polymerase II to IL-6 promoter in macrophages transfected with si-DHX9 or si-nc for 48 h and later incubated with 40 μg/mL oxLDL for 24 h. **e** ChIP analysis of the binding of p65 to IL-6 promoter in macrophages transfected with si-DHX9 or si-nc for 48 h and later incubated with 40 μg/mL oxLDL for 24 h. **f** and **g** ChIP-re-ChIP analysis of the binding of RNA Polymerase II-p65 complex to IL-6 promoter in macrophages transfected with si-DHX9 or si-nc for 48 h and later incubated with 40 μg/mL oxLDL for 24 h. Data are represented as means ± SD (n = 3; *P < 0.05).

Owing to that DHX9 is found to interact with p65 to form DHX9-p65 complex, which then links to RNA Polymerase II holoenzyme complex to regulating the transcriptional activity of NF-κB [30], we then examined whether oxLDL affects the transcriptional activity of DHX9-p65 complex. As shown in Fig. 4a, ChIP analysis showed that oxLDL stimulation significantly increased DHX9 binding to the promoter of IL-6. Furthermore, ChIP-re-ChIP analysis showed that oxLDL stimulation significantly increased DHX9-p65 complex binding to the promoter of IL-6 (Fig. 4b, c). Besides, as expected, knockdown of DHX9 significantly reduced RNA Polymerase II or P65 binding to the promoter of IL-6 (Fig. 4d, e). In addition, knockdown of DHX9 also significantly reduced RNA Polymerase II-P65 complex binding to the promoter of IL-6 (Fig. 4f, g). Collectively, these results suggest that oxLDL stimulation promotes the transcriptional activity of DHX9-p65-RNA Polymerase II complex.

### Knockdown of DHX9 Alleviates AS Progression *In Vivo*

**Fig. 5:**
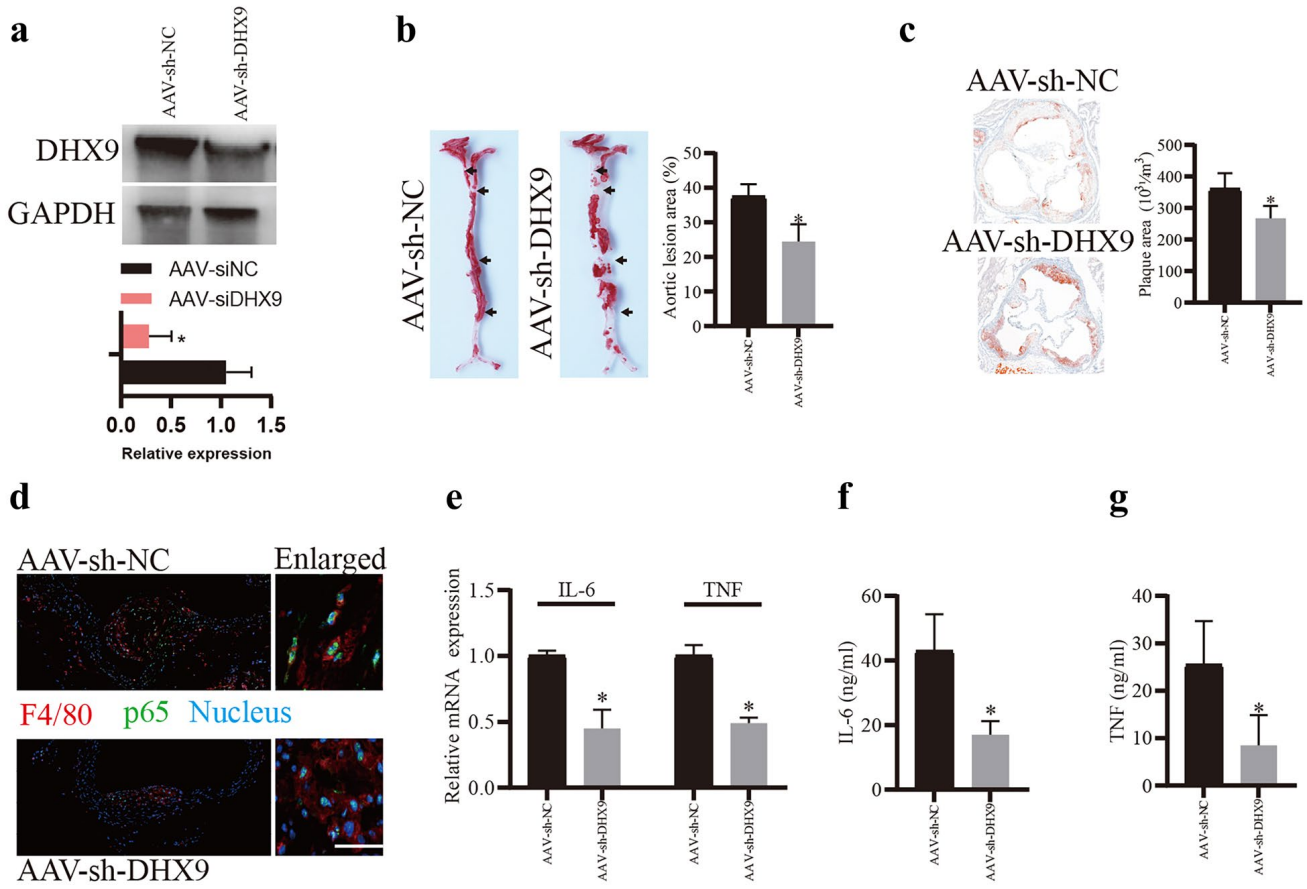
Knockdown of DHX9 alleviates AS progression *in vivo. ***a** Western blot analysis of DHX9 protein expression in the arterial tissues of AAV- sh-DHX9 group mice and AAV- sh-NC group mice. **b** Representative images and quantification of the aorta en face lesion stained with oil red O (n = 6 for each group). Data are represented as means ± SD (n = 6; *P < 0.05). **c** Representative images and quantification of the aortic root lesion area stained with oil red O (n = 6 for each group). Data are represented as means ± SD (n = 6; *P < 0.05). **d** Immunofluorescence analysis of F4/80 and p-p65 in the plaques of arteries of AAV- sh-DHX9 group mice and AAV- sh-NC group mice. Scale bars, 20 μm. **e** qPCR detection of IL-6 and TNF-α mRNA expressions in the plaques of arteries of AAV- sh-DHX9 group mice and AAV- sh-NC group mice. **g** and **f** ELISA detection of IL-6 and TNF-α expressions in the plasma of mice. Data are represented as means ± SD (n = 3; *P < 0.05).

To explore the role of DHX9 during AS progression *in vivo*, mice were injected with AAV-mediated shRNA-DHX9 or AAV-shRNA-control for 1 week through tail vein, respectively, and then fed with western diet (WD) for 12 weeks to establish AS model. As expected, DHX9 protein expression in the arterial tissues of AAV- sh-DHX9 group was significantly downregulated (Fig. 5a). Furthermore, the lesion areas in the aortic roots of AAV- sh-DHX9 group mice were significantly smaller than those of control mice (Fig. 5b). En face aorta analysis showed that the lesion area was significantly smaller in AAV-sh-DHX9 group mice (Fig. 5c). Besides, immunofluorescent staining found that knockdown of DHX9 significantly decreased p65 activation in the macrophages stained with F4/80 antibody in the plaques of arteries (Fig. 5d). Moreover, the mRNA expressions of IL-6 and TNF-α were also significantly decreased in the arterial tissues of AAV-sh-DHX9 group, compared to the control group (Fig. 5e). Finally, in the plasma of mice, ELISA assay showed that IL-6 and TNF-α expression levels in the plasma of AAV-sh-DHX9 group mice were also significantly decreased (Fig. 5f, g). Overall, these results indicate that knockdown of DHX9 alleviates AS progression *in vivo*.

### Knockdown of DHX9 Inhibits P65 Activation, Inflammatory Factor Expressions, and the Transcriptional Activity of P65-RNA Polymerase II Complex in PBMCs From CAD Patients

**Fig. 6:**
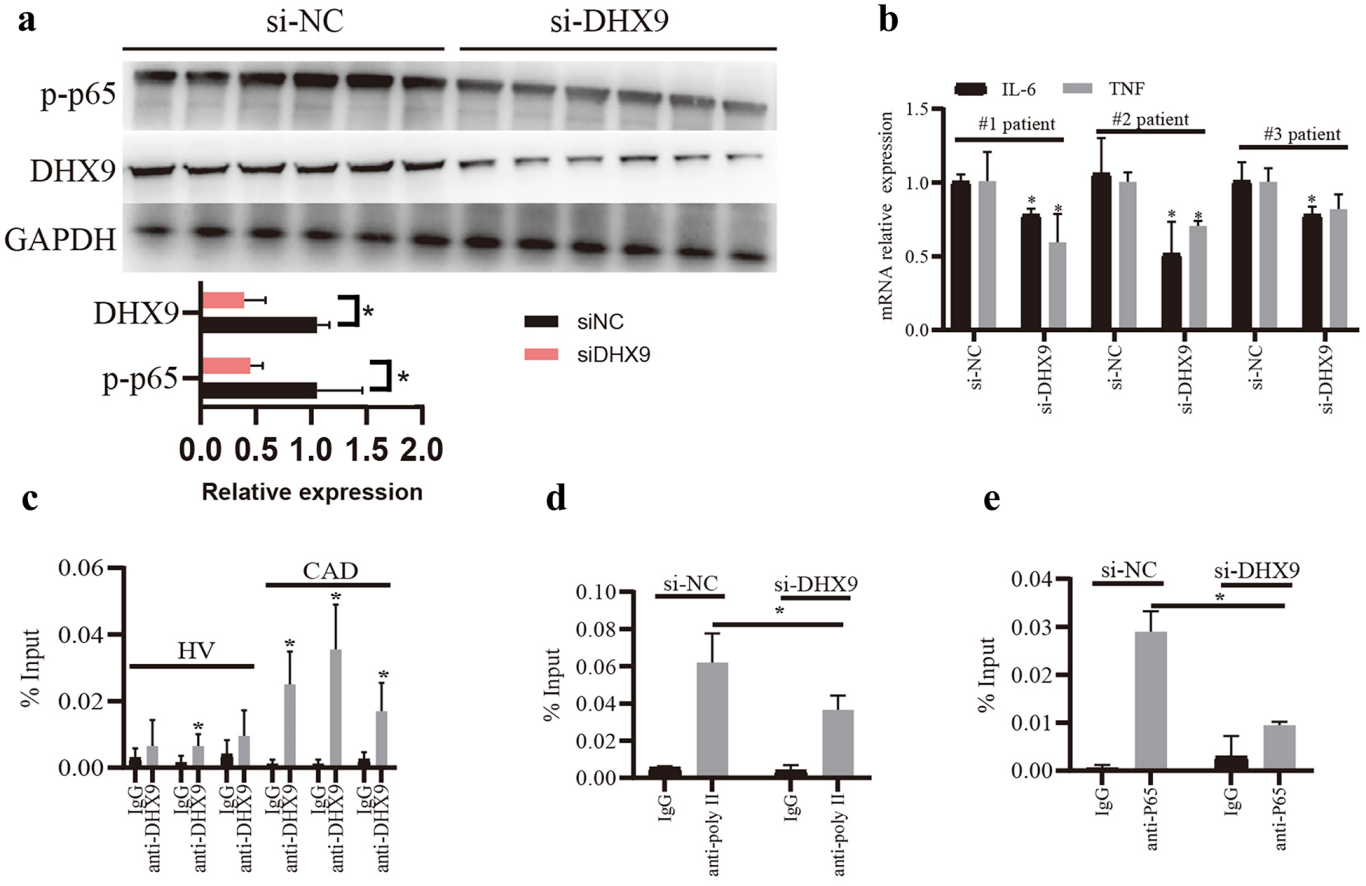
Knockdown of DHX9 inhibits p65 activation, inflammatory factor expressions, and the transcriptional activity of p65-RNA Polymerase II complex in PBMCs from CAD patients. **a** Western blot analysis of DHX-9 and p-p65 protein expressions in the PBMCs from CAD patients transfected with si-DHX9 or si-NC. **b** qPCR detection of IL-6 and TNF-α mRNA expressions in the PBMCs from CAD patients transfected with si-DHX9 or si-NC. **c** ChIP analysis of the binding of DHX9 to IL-6 promoter in PBMCs. **d** ChIP analysis of the binding of RNA Polymerase II to IL-6 promoter in the PBMCs from CAD patients transfected with si-DHX9 or si-NC. **e** ChIP analysis of the binding of p65 to IL-6 promoter in the PBMCs from CAD patients transfected with si-DHX9 or si-NC. Data are represented as means ± SD (n = 3; *P < 0.05).

Finally, we examined the effects of DHX9 on inflammatory response using PBMCs from patients with CAD. As shown in Fig. 6a, knockdown of DHX9 significantly decreased the p-p65 expression levels in the PBMCs from CAD patients. Furthermore, knockdown of DHX9 significantly decreased the mRNA expressions of IL-6 and TNF-α in the PBMCs from CAD patients (Fig. 6b). Besides, as expected, ChIP analysis showed that more DHX9 proteins were found to bind to the promoter of IL-6 in the PBMCs from CAD patients, compared to HV (Fig. 6c). Moreover, knockdown of DHX9 also significantly decreased RNA Polymerase II or P65 binding to the promoter of IL-6 in the PBMCs (Fig. 6d, e). Overall, these results indicate that knockdown of DHX9 inhibits p65 activation, inflammatory factor expressions, and the transcriptional activity of p65-RNA Polymerase II complex in PBMCs from CAD patients.

## DISCUSSION

**Fig. 7:**
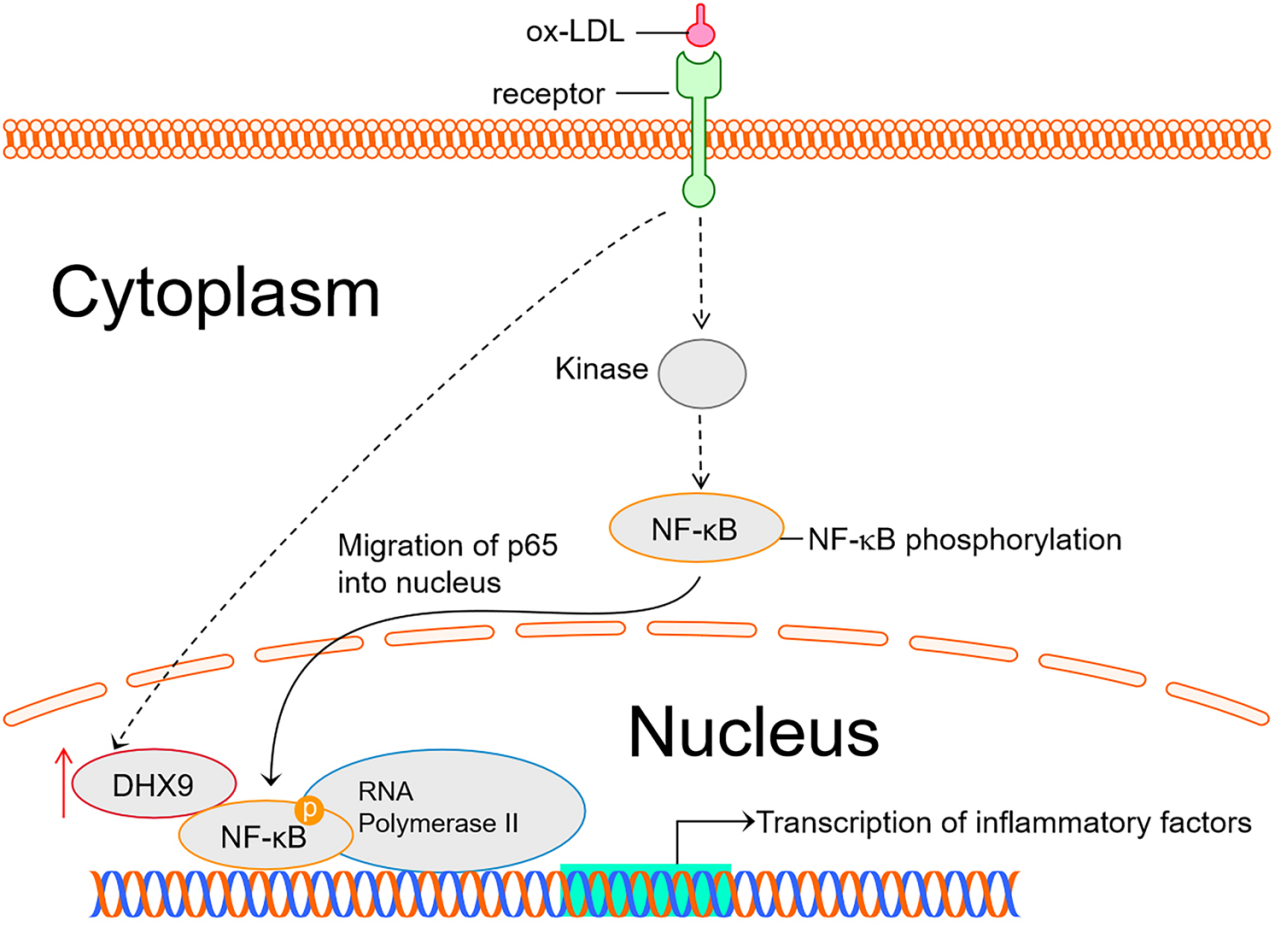
DHX9 promotes ox-LDL-induced inflammation in macrophages via interacting with p65. DHX9 interacts with p65 in ox-LDL-stimulated macrophages to enhance the transcriptional activity of DHX9-p65-RNA Polymerase II complex to produce inflammatory factors.

Atherosclerosis is a chronic inflammatory disease which is a major cause of cardiovascular disease [31]. Macrophage-mediated inflammatory response by plasma-derived lipoproteins and formation of lipid-filled foam cells play essential roles in the initiation of atherosclerotic lesion formation [32]. Thus, resolution of inflammation in macrophages has been proved to be an attractive method for alleviating AS, such as using omega-3 fatty acids or arachidonic acid [33, 34]. In this study, we report that DHX9, as an autoantigen in the sera of systemic lupus erythematosus patients, is highly expressed in the PBMCs from patients with CAD, and interacts with p65 in macrophages to enhance the transcriptional activity of DHX9-p65-RNA Polymerase II complex to produce inflammatory factors (Fig. 7). Knockdown of DHX9 could efficiently alleviate AS progression in ApoE -/- mice fed with western diet.

DHX9, as an ATP-dependent nucleic acid helicase, plays important roles in regulating DNA replication, RNA regulation and mRNA translation [35, 36]. In this study, we find that oxLDL stimulation promotes DHX9 interaction with p65 in nucleus to form DHX9-p65-RNA Polymerase II complex to enhance the transcription of inflammatory factors, which is consistent with recent study that nuclear DHX9 functions as a transcription coactivator in the stimulation of NF-κB-mediated innate immunity against DNA virus infection [36]. Besides inflammatory responses, AS is also reported to be closely associated with damage to the DNA of both circulating cells, and cells of the vessel wall [36, 37]. For example, patients with CAD have a higher micronucleus index (a marker of genetic instability) than healthy controls, which correlates with disease severity [38]. Recently, DHX9 has been found to promote R-loop formation in cells with impaired RNA splicing, and to function as a molecular player in R-loop-associated DNA damage [39]. Whether DXH9 is also involved in DNA damage during AS development needs to be further investigated.

Adhesion of monocytes to micro-injuries on arterial walls plays an important role in the early stage of AS development [40]. Some studies report that the adhesive ability of monocytes to the endothelial cells or collagen type I, a major element of normal arterial wall matrix and atherosclerotic plaques, are evidently elevated in old individuals, compared to young individuals [41, 42], probably owing to that differentially expressed adhesion molecules on monocytes between older and young individuals [42]. In this study, we find that knockdown of DHX9 significantly ameliorates TNF-α-mediated monocyte adhesion to HUVECs. However, DHX9 has been found to slow aging probably through regulating p53-dependent growth arrest and premature senescence [43]. Whether DHX9 affects the phenotypic and functional of circulating monocytes, especially the adhesion molecules expressions, needs to investigated in our following studies.

Overall, our study firstly finds that DHX9 promotes AS progression by enhancing inflammation in macrophages through enhancing the transcriptional activity of DHX9-p65-RNA Polymerase II complex, and suggests DHX9 as a potential target for developing therapeutic drug.

## Data Availability

All data generated and/or analyzed during this study are included in this published article.

## Abbreviations

AS: Atherosclerosis
DHX9: DExH-Box helicase 9
PBMCs: Peripheral blood mononuclear cells
CAD: Coronary artery disease
LDL: Low-density lipoprotein
ox-LDL: Oxidized LDL
EGFR: Epidermal growth factor receptor
MARCH: Membrane-associated ring-CH-type finger
MAVS: Mitochondrial antiviral signaling protein
HUVECs: Human umbilical vein endothelial cells
HV: Healthy volunteers
siRNAs: Small interfering RNAs
ELISA: Enzyme-linked immunosorbent assay
Co-IP: Protein immunoprecipitation
ChIP: Chromatin immunoprecipitation
GM-CSF: Granulocyte-macrophage colony-stimulating factor
IFN-γ: Interferon-γ
STAT1: Signal transducer and activator of transcription 1
MAPK: Mitogen-activated protein kinase
JNK: Jun-NH(2)-terminal kinase
ERK: Extracellular signal-regulated kinase
NF-κB: Nuclear factor kappa-light-chain-enhancer of activated B cells
WD: Western diet
